# Supplementary material for: Sample, time, and wording effects on estimating the prevalence of childfree adults: Insights from Japan
Source: PLoS One. 2024 Apr 16;19(4):e0302184. doi: 10.1371/journal.pone.0302184 (PMC11020949; doi:10.1371/journal.pone.0302184)
Supplement: Attachment — Submitted filename: Response to reviewers.pdf [file pone.0302184.s001.pdf]

Summary of revisions to:
Sample, time, and wording effects on estimating the prevalence of childfree adults: Insights from Japan (PONE-D-23-35738)

Dear Dr. Kelly (editor) and Dr. Jin (reviewer),

Thank you for your very helpful and constructive comments, and for the opportunity to revise and resubmit our paper. We have revised our manuscript to address your concerns, and as a result, we believe the new version of the manuscript is improved. In this letter, we include point-by-point responses to the reviewer's comments.

1 Dr. Chuyao Jin (Reviewer #1)

1.1 "Thanks for the opportunity to review this well-written paper. This paper set the scene for future studies on how to estimate and report the prevalence of childfree adults. However, some aspects should be considered before accepting this paper."

We are happy to hear that you found the paper well-written, and agree that it offers some useful guidance for future studies aiming to estimate childfree prevalence. We appreciate your helpful comments, which we address below.

1.2 "Although the authors gave a clear definition of "childfree adults" (i.e., adults who neither have nor want biological, adopted, foster, or step-children), some cited literature [1] only considered biological children when they defined different types of childlessness, which is inconsistent with the authors' definition. I suggest the authors to double check how "childfree" was defined in their citations, and only cite those using the same definition or clarify the differences in the definition. Also, for the question wordings presented in Table 1, it may not be appropriate to assume that "children" equals to "both biological and non-biological children". Although not explicitly stated, the questions like "do you want to have children" or "do you plan to have children in the future" may imply a focus on biological children, as the authors commented in another paper [2]. The authors should discuss these nuances unless the Japanese surveys explicitly define children as both biological and non-biological."

In this revised version, for the purposes of this study, we now define a childfree adult as adults who "*neither have nor want children*". This definition most closely matches the questions used to collect data for the surveys we meta-analyze, which did not explicitly indicate whether responses should include only biological children, or both biological and non-biological children. Additionally, in the discussion section we now identify this as a limitation and direction for future research: "*although definitions of 'childfree' vary in whether they are restricted to biological children [1] or both biological and non-biological (e.g., adopted, foster, and step-) children [2], we are unable to test whether such differences in definition affect prevalence estimates because the questions on these surveys do not explicitly indicate whether 'children' includes only biological children, or both biological and non-biological. It is rare for adults to have only non-biological children, so the scope of the definition likely has limited effect on the prevalence estimates of childfree adults. However, future studies should explore the potential effect.*"

1.3 "It would be helpful if the authors could provide the demographic characteristics and sampling methods of the five national surveys and raw data of the 83 estimates."

We now provide detailed information about each survey's sampling methods in the *Supplementary Information* available at `https://osf.io/wkeqp/`. Each of these survey was nationally representative, which means that the demographic characteristics of each survey sample matches the demographic characteristics of the national population. We have access only to the gender, marital status, and age range of each survey sample; we report this information for each of the 83 samples in the *Supplementary Information*. We do not have access to other demographic information about these samples.

The raw data for our meta-analysis are the 83 prevalence estimates extracted from government surveys. These extracted prevalence estimates, as well as the numerator and denominator from which they are constructed, are provided in the file called `cf_in_japan.R`, which is available from our project repository at `https://osf.io/wkeqp/`. In addition, the entire raw data from which the meta-regression is estimated are displayed as Table S1 in the *Supplementary Information*.

1.4 "Do all the 83 estimates come from populations aged 18 to 50, or are some from younger (e.g., 18-30) and some from older (e.g., 30-50) age groups? Different age ranges may confound the results."

Thanks for this very helpful observation! In the revised paper, we have expanded our analysis to explicitly account for the potential confounding effect of differing age ranges across the samples from which these 83 estimates were obtained. In the methods section, we now explain: "*Some estimates were computed from samples with a wide age range (e.g., JNFS in 2005, ages 18-50), while others were computed from samples with narrower age ranges that are younger (e.g., LSA12 in 2013, ages 21-30) or older (e.g., LSA02 in 2011, ages 30-44). To account for the variation in age ranges across samples, we control for each sample's minimum and maximum age, centering these variables on 18 and 50, respectively.*" The inclusion of these statistical controls did not change our results or conclusions.

1.5 "The authors mentioned "A forest plot summarizing every included prevalence estimate is available in the supplementary materials", but I didn't find any supplementary materials."

We apologize for the difficulty in locating the supplementary materials, which are located in the project's repository at `https://osf.io/wkeqp/`. This link is provided in the last sentence of the Methods section in the manuscript, and in the paper's Data Availability Statement. This repository contains the data and code necessary to replicate our findings, as well as the *Supplementary Information*. The *Supplementary Information* formerly contained a forest plot. However, we have replaced it with a table because it is easier to read and contains more information.

References

[1] Abma JC, Martinez GM. Childlessness among older women in the United States: Trends and profiles. Journal of Marriage and Family. 2006;68(4):1045–1056. doi:10.1111/j.1741-3737.2006.00312.x.

[2] Neal ZP, Neal JW. A framework for studying adults who neither have nor want children. The Family Journal. 2023;doi:10.1177/10664807231198869.
