# Supplementary material for: Sample, time, and wording effects on estimating the prevalence of childfree adults: Insights from Japan
Source: PLoS One. 2024 Apr 16;19(4):e0302184. doi: 10.1371/journal.pone.0302184 (PMC11020949; doi:10.1371/journal.pone.0302184)
Supplement: Attachment — Submitted filename: comments V2.docx [file pone.0302184.s002.docx]

Thanks for providing detailed information on the analysis and addressing all my comments. I still have two questions.

Firstly, it seems a bit unusual that the authors adjusted for the minimum and maximum age to address the effects of age. Using only min and max values may not accurately represent the distribution of age. For instance, a study with one hundred 18-year-olds and one 50-year-old, and another study with one 18-year-old and one hundred 50-year-olds would yield the same minimum and maximum ages. I would suggest two alternative approaches for handling age:

1) Use the mean or median age; or
2) Create a categorical variable, such as young group (e.g., 18-30), old group (e.g., 30-50), and all-ages group (e.g., 18-50).

Secondly, I am curious about the changes in sample size and estimated prevalence of the childfree population in the revised version. The sample size decreased from 4 million to 2.5 million, and the prevalence dropped from 8.83% to 5.64%. Could you clarify whether these changes are due to typos in the previous version or if the authors modified the inclusion and exclusion criteria?
